# Ten Simple Rules for Developing Usable Software in Computational Biology

**DOI:** 10.1371/journal.pcbi.1005265

**Published:** 2017-01-05

**Authors:** Markus List, Peter Ebert, Felipe Albrecht

**Affiliations:** 1 Computational Biology and Applied Algorithmics, Max Planck Institute for Informatics, Saarland Informatics Campus, Saarbrücken, Germany; 2 Graduate School of Computer Science, Saarland Informatics Campus, Saarbrücken, Germany; Dassault Systemes BIOVIA, UNITED STATES

## Introduction

The rise of high-throughput technologies in molecular biology has led to a massive amount of publicly available data. While computational method development has been a cornerstone of biomedical research for decades, the rapid technological progress in the wet lab makes it difficult for software development to keep pace. Wet lab scientists rely heavily on computational methods, especially since more research is now performed in silico. However, suitable tools do not always exist, and not everyone has the skills to write complex software. Computational biologists are required to close this gap, but they often lack formal training in software engineering. To alleviate this, several related challenges have been previously addressed in the Ten Simple Rules series, including reproducibility [1], effectiveness [2], and open-source development of software [3, 4].

Here, we want to shed light on issues concerning software usability. Usability is commonly defined as “a measure of interface quality that refers to the effectiveness, efficiency, and satisfaction with which users can perform tasks with a tool” [5]. Considering the subjective nature of this topic, a broad consensus may be hard to achieve. Nevertheless, good usability is imperative for achieving wide acceptance of a software tool in the community. In many cases, academic software starts out as a prototype that solves one specific task and is not geared for a larger user group. As soon as the developer realizes that the complexity of the problems solved by the software could make it widely applicable, the software will grow to meet the new demands. At least by this point, if not sooner, usability should become a priority. Unfortunately, efforts in scientific software development are constrained by limited funding, time, and rapid turnover of group members. As a result, scientific software is often poorly documented, non-intuitive, non-robust with regards to input data and parameters, and hard to install. For many use cases, there is a plethora of tools that appear very similar and make it difficult for the user to select the one that best fits their needs. Not surprisingly, a substantial fraction of these tools are probably abandonware; i.e., these are no longer actively developed or supported in spite of their potential value to the scientific community.

To our knowledge, software development as part of scientific research is usually carried out by individuals or small teams with no more than two or three members. Hence, the responsibility of designing, implementing, testing, and documenting the code rests on few shoulders. Additionally, there is pressure to produce publishable results or, at least, to contribute analysis work to ongoing projects. Consequently, academic software is typically released as a prototype. We acknowledge that such a tool cannot adhere to and should not be judged by the standards that we take for granted for production grade software. However, widespread use of a tool is typically in the interest of a researcher. To this end, we propose ten simple rules that, in our experience, have a considerable impact on improving usability of scientific software.

## Rule 1: Identify the Missing Pieces

Unless you are a pioneer, and few of us are, the problem you are working on is likely addressed by existing tools. As a professional, you are aware of this software but may consider it cumbersome, non-functional, or otherwise unacceptable for your demands. Make sure that your judgment is shared by a substantial fraction of the prospective users before you start developing a new tool. Usable software should offer the features needed and behave as expected by the community. Moreover, a new tool needs to provide substantial novelty over existing solutions. For this purpose, list the requirements on the software and create a comparison table to set the new tool against existing solutions. This allows you to carve out the selling points of your tool in a systematic fashion.

## Rule 2: Collect Feedback from Prospective Users

Software can be regarded as providing the interface between wet lab science and data analysis. A lack of communication between both sides will lead to misunderstandings that need to be rectified by substantially changing the code base in a late phase of the project. Avoid this pitfall by exposing potential users to a prototype. Discussions on data formats or on the design of the user interface will reveal unforeseen challenges and help to determine if a tool is sufficiently intuitive [6]. To plan your progress, keep a record of suggested improvements and existing issues.

## Rule 3: Be Ready for Data Growth

First estimate the expected data growth in your field and then design your software accordingly. To this end, consider parallelization and make sure your tool can be integrated seamlessly in workflow management systems (e.g., GALAXY [7] and Taverna [8]), pipeline frameworks (e.g., Ruffus [9] and SnakeMake [10]), or a cluster framework (e.g., Hadoop, http://hadoop.apache.org/). Moreover, make sure that the user interface can scale to growing data volumes. For example, consider that the visualizations should still be comprehensible for larger datasets, e.g., by displaying only parts of the data or through aggregation of results.

## Rule 4: Use Standard Data Formats for Input and Output

As an expert in your research domain, you know the established data standards and related programming libraries for reading and writing commonly used data formats. Make sure that your tool’s output follows standard specifications to the letter, but be as lenient as possible when users provide non-standard input. Tools that follow this rule are more likely to become successful. If you are working in an emerging field with no prevalent model for data exchange, provide data in a structured text file (e.g., tab-separated tables, XML/XSD, or JSON) and aim for self-documenting output by including header lines and data type descriptions. In this case, document how users can derive suitable input data for your tool.

## Rule 5: Expose Only Mandatory Parameters

Exposing all (possible) parameters to a user can be confusing and carries the risk of nonsensical parameters settings. When possible, users will thus rely on default parameters. The same applies to benchmark studies comparing your tool against the state-of-the-art competitors. This has three important implications: (i) expose only a small set of parameters by default whose effects on results can be easily understood by any user, (ii) offer advanced parameters only in an expert section and describe them thoroughly in the documentation, and (iii) choose conservatively (and if possible, justify) the default values for parameters such that the tool can operate in a wide range of scenarios and within reasonable run time.

## Rule 6: Expect Users to Make Mistakes

You should never assume that your tool is self-explanatory, that requirements concerning the input data are obvious, or that the user will immediately grasp all details of the problem at hand. Ideally, your tool supports the user in using it appropriately, e.g., by checking that data remain inside required ranges or that identifiers are unique, and provides descriptive error messages in case of unexpected values. If performance penalties due to such checks are a real concern (which should be tested), make the checks optional and enabled by default. Finally, allow users to stop ongoing operations in case they realize they made a mistake.

## Rule 7: Provide Logging Information

Two types of logs improve usability and also support the user in making their research more reproducible. Configuration logs keep track of basic information, such as the time stamp of the analysis, the version of your tool and of third-party libraries, as well as the parameter settings and input data. Archiving this information is particularly important in long-running research projects in order to trace irregularities in the results at any later point in time [1]. Technical logs, on the other hand, contain progress messages that help users to pinpoint errors in the execution flow and allow clear communication of these issues to the developer. As much as possible, avoid exposing potentially sensitive user information in the logs.

## Rule 8: Get Users Started Quickly

Complex setup routines introduce dependency [11] or configuration debt [12]; i.e., the user has to spend substantial time installing software and learning about the execution parameters of a tool. These raise the bar for unhindered exploration of software features. Such issues can be solved by implementing a web application (if feasible with respect to resource demands), by providing a standalone executable, or by providing a system-specific software package. Alternatively, issues of a program’s dependence on third-party libraries can be avoided by encapsulating your tool in a virtual machine image or, e.g., a Docker container (https://docker.com). Finally, it is imperative to provide demo data that enable users to immediately interact with the software. A successful test run proves to the user that your software works as expected and will be essential if you want your tool to be published.

## Rule 9: Offer Tutorial Material

Researchers can seldom afford the time to thoroughly read complex user manuals. They will thus appreciate a number of clearly written code examples, illustrations, or video screen casts to get started. Most importantly, documented use cases enable users to quickly assess if your tool is suited for the problem at hand and allow fast learning by doing. Keep in mind that these materials have to be updated together with your tool.

## Rule 10: Consider the Future of Your Tool

For long-term availability of your software, use suitable repositories such as github (https://github.com) or bitbucket (https://bitbucket.com) throughout the development process. Explicitly state under which software license you release your code for third parties (see https://opensource.org/licenses). Without such a license, using your software might be prohibitive for many organizations or companies. More importantly, keeping your code in a public repository will also allow you to engage with the users through issue tracking (e.g., bugs, suggestions). After releasing your tool, expect support requests and take them seriously. See them as an opportunity to continuously improve the usability of your tool.

## Conclusions

Usability is an important topic in software design, and we would like to provide a few starting points for further reading [13–18]. In the above ten simple rules, we highlight that software should not only be scientifically sound but also be perceived as usable for widespread and effective application. To these ends, developers should also be the first to apply their tool, to reveal usability issues as early as possible. However, effort is required from both users and developers to further improve a tool. Even engaging with only a few users (Rule 2) is likely to have a large impact on usability, since, as Jakob Nielsen put it, “Zero users give zero insights” [19].
